# Agency and reward across development and in autism: A free-choice paradigm

**DOI:** 10.1371/journal.pone.0284407

**Published:** 2023-04-12

**Authors:** Irene Valori, Laura Carnevali, Teresa Farroni

**Affiliations:** 1 Department of Developmental Psychology and Socialisation, University of Padova, Padova, Italy; 2 Centre for Tactile Internet with Human-in-the-Loop (CETI), Chair of Acoustic and Haptic Engineering, Technische Universitat Dresden, Dresden, Germany; Kyoto University Graduate School of Informatics: Kyoto Daigaku Daigakuin Johogaku Kenkyuka, JAPAN

## Abstract

Our ability to perform voluntary actions and make choices is shaped by the motivation from control over the resulting effects (agency) and from positive outcomes (reward). The underlying action-outcome binding mechanisms rely on sensorimotor abilities that specialise through child development and undergo different trajectories in autism. The study aimed at disentangling the role of agency and reward in driving action selection of autistic and non-autistic children and adults, who were asked to freely select one of three candies and feed the animals appearing on a tablet. The candies were associated with different probabilities of delivering a neutral vs no effect (agency task), or a positive vs neutral effect (reward task). Choices and reaction times (RT) were measured to understand whether participants preferred and were faster at selecting options with higher probability of producing a neutral vs. no effect (agency) or a positive vs. neutral effect (reward). Participants’ choices and RT were not affected by agency, whereas a more frequent selection of the option with higher probability of a positive vs. neutral effect emerged across groups, thus suggesting a reward effect. Autistic participants selected less frequently the option with chance level of receiving a neutral or no effect, which could be interpreted as a sign of reduced tolerance of uncertainty. Across tasks, conditions and age groups, autistic participants presented shorter RT, which is a marker of reduced action planning and control. Future research should deepen how tolerance of uncertainty, action planning and control impact the way autistic individuals make choices in everyday life situations, potentially contributing to restricted and repetitive behaviours.

## Introduction

In everyday life, we perform voluntary, goal-oriented actions for which we hold ourselves responsible. The sense of agency can be defined as the perception of control over one’s own actions and the external world and can be traced back to the ability to recognize oneself as the cause of an event [1–3]. Beyond the explicit and conscious recognition of oneself as the cause of an event, implicit agency arises from the sensorimotor match between the expected and actual outcomes of one’s action. It is built upon sensory-sensory and sensory-motor congruency and disrupted by action-effect delays [4,5].

One of the most widely used implicit measures of agency is the intentional binding effect that consists in the tendency of agents to perceive the time interval between a voluntary action and a sensory stimulus as shorter than it actually is. More specifically, the onset of the voluntary action is reported later in time and awareness of the sensory feedback is temporally anticipated [5]. Recently, the Control-Based Response Selection framework (CBRS) proposed that producing effects that are perceived as self-caused facilitates action selection and execution. People more frequently and faster select response options associated with higher probability of producing an effect, compared to no effect, thus being implicitly motivated and facilitated by the associated sense of control [6,7]. In everyday life, it could be that fluently selecting an action makes it more likely that our intentions will be realized, and the expected outcome achieved. Individuals report greater perceptions of control over a given event when prime stimuli allow for more fluent and immediate action selection [8]. According to this, habitual actions are accompanied by a strong sense of control and could be therefore sustained by agency mechanisms. Intriguingly, the motor system might be insensitive to abstract representations of the valence of an effect (i.e., receiving a positive rather than neutral effect does not speed the action up) [9].

However, our actions are certainly shaped by the valence of their consequences. We prefer to perform actions associated with positive effects, which have a motivational value and can be defined as rewards. Research has extensively investigated agency and reward as separate mechanisms, showing that they contribute differently to people’s action selection, shape distinct aspects of behaviour and emerge from distinct neural bases [10]. On the contrary, agency and reward are closely interconnected during naturalistic interactions with the outside world. Indeed, when an action that the agent perceives as voluntary has a consequence that is interpreted as self-caused and also positive, the two experiences are concomitant. For instance, people are biased in attributing positive outcomes to themselves [11], suggesting that the motivation derived from the sense of agency and that derived from the positive valence of the outcome are indeed interconnected. Implicit agency increases when people have a higher number of alternatives to select, they can make free rather than instructed choices, the outcome is positive in valence [12,13]. Other researchers found that positive outcomes retrospectively enhance implicit agency, which is particularly true when the outcome valence is unexpected or unpredictable [14].

Neural evidence suggests that agency and reward might act similarly in facilitating people’s selection of actions, specifically influencing the motor planning phase [10]. Preparatory neural activity in motor and premotor areas anticipates voluntary movements and contributes to agency [15], which results in a sense of control that makes actions faster [7]. Similarly, there is evidence that monetary rewards make actions faster, with reward magnitude being associated with the activation of motor and pre-motor brain areas, potentially promoting motor planning prior to action execution [16]. In addition, the reward system activation increases when individuals receive self-caused vs random rewards [17]. This evidence suggests that agency might modulate the way rewards are processed and promote learning.

Throughout child development, the mechanisms underlying and associated with agency and reward are subject to specialization and tuning and may undergo atypical trajectories under specific neurodevelopmental conditions. Notably, the thresholds for detecting temporal biases between action and consequence change during development. From the age of 4 to 15, there is a progressive decrease in the minimum temporal delay necessary for the person to be aware of the action-effect alteration [18]. Overall, the temporal interval within which multisensory stimuli are likely to be perceptually bound (namely, multisensory temporal binding window) gradually decreases up to adolescence [19]. Multisensory development goes hand-in-hand with motor development, in a perception-action cycle that allows the individual to learn from their actions [20]. Infants learn through embodied sensorimotor contingencies, thus using their bodies to produce effects in the external world [21,22] and responding faster to events that they previously had actively produced than to action-independent events [23]. It has been argued that the mere association between stimulus and response is not sufficient to constitute minimal sense of agency, which should be distinguished from reinforced learning [24]. On the other hand, the two processes are often concomitant and probably modulate each other. For instance, toddlers orient their attention toward stimuli that respond to their gaze, with a preference for social compared to non-social stimuli [25]. It is not yet clear how the child’s sensorimotor progress is linked to their sensitivity to implicit agency and reward. Some authors found reduced temporal binding in school-aged children compared with adults [26,27], while other authors found adult-levels of intentional binding in children from 6 years of age [28].

The sensorimotor functioning needed to capture action-effect correspondences and experience agency and reward varies under conditions of neurodiversity [29]. People on the autism spectrum show broad differences at the multisensory level [30,31], with multisensory facilitation and higher reliance on unimodal processing [32], an extended multisensory temporal binding window [33], reduced integration of multimodal (e.g., audio-visual) cues [34], different integration of interoceptive and exteroceptive stimuli [35]. They frequently manifest difficulties in action planning, monitoring and prediction [36,37]. At the neural level, children on the autism spectrum showed reduced event-related desynchronization before movements [38] and altered movement related potential during action planning [39], which are interpreted as markers of reduced motor preparation. Moreover, altered neural activity during response monitoring was correlated with repetitive behaviours [40] and social difficulties [41] in autistic people. Ineffective motor planning seems to be associated with motor stereotypies [42], which are involuntary, restricted, and repetitive patterns of movements that limit the individual’s resources to learn and practice various, purposeful actions [43,44]. Notably, motor-related cortical potentials in premotor areas, which anticipate voluntary motor actions, are found to be absent before stereotypy onset [42].

Restrictive and repetitive behaviours (among which stereotypies) are one of the two macro-areas of symptoms for the diagnosis of autism [45]. They might come along with atypical action selection processes, among which agency and reward play a crucial role. Understanding these mechanisms in autism might shed light on how to promote not only learning but also volition and self-determination. Using implicit measurements of agency, some researchers found differences in the autistic adult population. Participants were asked to press the spacebar whenever they wanted. Sensory feedback was presented after a variable temporal delay (i.e., 250, 450, or 650 ms), and participants were required to estimate the delay. Despite being overall accurate in time perception, autistic adults showed reduced intentional binding compared to controls [37]. One may ask whether the enlarged multisensory temporal binding windows in autism [33] constitute an underlying barrier to the emergence of implicit agency.

To the best of our knowledge, there are no previous studies investigating agency in children on the autism spectrum, thus preventing us from understanding the developmental trajectory leading to any differences we can find in adult populations. The lack of knowledge on implicit agency across different age groups and neurodiverse populations is probably related to the fact that the most popular experimental paradigm (i.e., intentional binding) is only usable with people who have good verbal skills and understanding of abstract concepts, thus limiting their appropriateness for young children and people with difficulties in verbal communication and abstract reasoning. Other recently proposed paradigms are based on simple tasks of choosing between options and measuring the frequency of choices and kinematic parameters [9]. However, it remains to be clarified whether these tasks can be used with populations other than neurotypical adults.

Differences in autism were also found in the processing of the valence of the action outcome and external feedback. Neuroimaging research showed atypical activation of the reward system, suggesting a reduced motivation from rewards [46,47], which is particularly evident with respect to social rewards [48–51]. There is still no evidence in the literature that clarifies the link between multisensory and motor planning difficulties and the sensitivity to agency and reward in autistic people. We can hypothesise an association between differences in low-level sensorimotor action-outcome binding and motor planning, that are pivotal for experiencing agency and reward. We therefore hypothesise reduced agency and reward sensitivity in autism, that may ultimately result in less opportunities for self-driven learning.

### Aim and hypotheses

The present study aimed at investigating the role of agency and reward in driving people’s free choices across the lifespan and exploring potential differences in autism. Frequency of choices and reaction times have been measured while children and adults performed free-choice tasks whereby the probability of causing an effect and the effect valence (i.e., neutral or positive) were manipulated. We aimed at distinguishing the role of implicit agency (i.e., causing a neutral effect compared to no effect at all) and reward (i.e., causing a positive compared to a neutral effect) on action selection. We expect both agency and reward to increase the frequency of choices, and affect motor parameters of participants’ choices, thus reducing the action time (i.e., RT). We expect adults to be faster in selecting response options with higher probability of causing a neutral (vs. no effect), or a positive (vs. neutral) effect. We also aim to explore developmental differences among autistic and non-autistic children and adults. Younger children and autistic individuals may be less sensitive to the agency effect, given their less refined sensorimotor integration system. Non-autistic children may be also more sensitive than adults to the outcome valence (reward) and prefer the response options with higher probability of delivering a positive vs. neutral effect. Autistic individuals may be less driven by the positive effect, with no preference for the response options with higher probability of delivering a positive vs. neutral effect.

## Materials and methods

### Procedure

Adult participants and children’s parents signed a written consent form before taking part in the experiment, which received ethical approval from the Research Ethics Committee of the School of Psychology, University of Padova (protocol no. 3251). The experiment was carried out in accordance with the approved guidelines and regulations.

Participants sat on a desk and were free to play with a reversible laptop with a touchscreen that was set to tablet mode (Lenovo Yoga, 14” IPS Full HD 1920 x 1080, Intel® Core™-U i7). The experimental paradigm consisted of two tasks: agency and reward. On the initial screen of both tasks, participants were presented with three geometric figures positioned in the lower part of the display. After a random temporal delay (ranging from 500 to 1200 ms), a cue stimulus (one of 8 different animals) appeared in the upper central part of the screen. Participants were instructed to freely select and press one of the three geometric figures, as they were candies to feed the animal. As the task is designed to be feasible for people with limited verbal communication skills, in the event that a participant did not communicate verbally, the task was presented without verbal instructions. In this case, the experimenter showed the participant that when an animal appeared, one of the 3 candies could be clicked on. The experimenter demonstrated the game with 3 trials: clicking each of the 3 response options in sequence, from left to right. If a participant was not engaged in this demonstration, the touchscreen was simply made available to the participant to explore freely.

We manipulated the probability of the different response keys to deliver different effects. Crucially, no mention of the effects was made in the instructions, so obtaining or not obtaining the effect was not posited to participants as a purpose nor informed them regarding their performance. The tasks were therefore designed to tap on implicit mechanisms of action selection, which was meant to be driven by the action effect, even beyond explicit judgements, beliefs, or intentions. The paradigm was adapted from that used in Karsh and Eitam’s [7] study that investigated motivation from control (agency) in neurotypical adults. The advantage of this task is that participants simply make free choices between response options (keyboard keys) when a central stimulus appears. The use of minimal verbal instructions also makes it usable with young children and autistic individuals, even with minimal verbal skills or understanding of abstract concepts such as time (necessary in the intentional binding paradigms most commonly used to study implicit agency). Moreover, paradigms that allow for free choices among alternatives are known to elicit greater experience of agency than tasks with instructed actions and no alternatives [12,52].

### Agency task

Participant’s response was followed by either the appearance of a neutral effect (the animal immediately disappeared, and a black and white circle became visible for 150 ms), or no effect (the animal immediately disappeared, and nothing is shown for the next 150 ms). Each response key was associated with a different probability (10%, 50% and 90%) of providing the neutral effect.

### Reward task

Participant’s response was followed by either the appearance of a positive effect (the animal immediately disappeared, and a black and white smiley became visible for 150 ms), or a neutral effect (the animal immediately disappeared, and a black and white circle became visible for 150 ms). Each response key was associated with a different probability (10%, 50% and 90%) of providing the positive effect.

**Fig 1:**
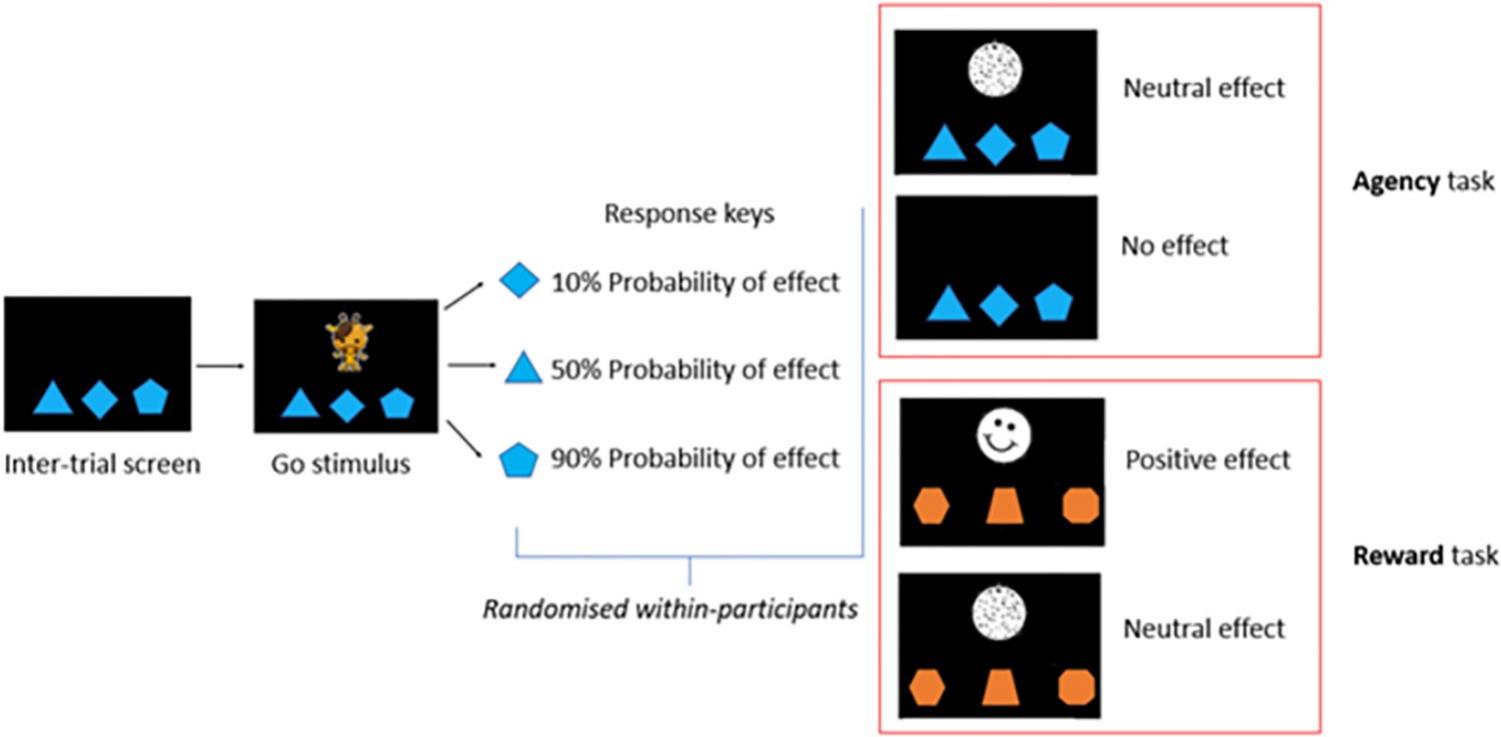
Agency and reward tasks.

A schematic representation of the agency and reward task is depicted in Fig 1.

The smiley is meant to have an intrinsic positive value, as it conveys positive, albeit very simplified, social information. The neutral effect has been built from the positive one, so that they have the same degree of black-white percentage to make them equally visible. The combination of shape, position on the screen and probability associated with the response keys was randomised between participants. Within participants, the shape and colour of the response keys varied from the agency to reward task, whereas the relative position (left, centre, right) of each probability (10%, 50% and 90%) remained constant. Participants were instructed to reply as quickly as possible. Failure to press any keys within 2,000 ms was marked as “omission” and a drawing of a hand appeared, which had to be clicked to move to the next trial. This caveat allowed the experimenter to briefly pause the task if the participant needed a break. The next trial started after a random delay (ranging from 500 to 1200 ms), which prevented participants from anticipating the onset of the next trial.

All participants firstly performed the agency task, and then the reward task, to avoid carryover effects (i.e., a potential reduction of the value of the neutral effect after receiving a positive effect in the previous block of trials). Each task ended upon completion of 104 total trials. The experimental session lasted about 20 minutes.

### Participants

Data collection took place between September 2021 and July 2022, as part of a collaborative project with several centres for autism in northern Italy, which offer various services in support of people on the autism spectrum and their families. All regular visitors to the centres were offered voluntary participation in the study. The final sample of children and adults on the autism spectrum was determined by the number of parents, children and adults who joined and participated. Psychologists confirmed participants’ diagnosis. Autistic participants carried out the experiment at their local centre. A convenient control group of children and adults with typical development in the same age range was tested at the University of Padova. Participants in the comparison group were not recruited on the basis of age range alone, but matched one-to-one with the autistic participants. In this way, despite the breadth of the three age groups, the autistic and non-autistic groups consist of pairings of 2 participants with an age difference of less than 2 years. According to parent-reports, typically developing children had no medical nor neuropsychological conditions. According to self-reports, typically developing adults had no medical nor neuropsychological conditions. Participants older than 16 years of age completed the Autism-spectrum Quotient—AQ questionnaire to assess the presence of autistic traits [53]). Since autism is an inherently heterogeneous condition, we have not established inclusion or exclusion criteria based on IQ, level of support needed, or possible presence of co-occurring medical or neuropsychological conditions. Thus, we aimed to include participants along the whole spectrum.

Our sample constitutes of 55 autistic participants and 55 neurotypical participants, across 3 age groups: younger children (from 6 to 10 years of age), older children (from 11 to 16 years of age), and adults (from 17 to 35 years of age). We have selected these age ranges in the light of the extant knowledge of discontinuous cognitive development between school age, adolescence, and adulthood. Specifically, between the ages of 10–11 and 16–17, there is an increase in reaction times to certain cognitive tasks, commonly attributed to the processes of synaptic proliferation and pruning that occur at puberty and adolescence [54].

We established to exclude participants who would have demonstrated (verbally or non-verbally) that they were unwilling or unable to continue until the end of the tasks (with less than 50% valid trials in each task), and/or never selected some of the response options, thus being unable to learn its action-effect characteristics. Five additional participants (n = 3 autistic, n = 2 non-autistic) were tested but have not been included in the final sample due to inability/unwillingness to perform the task. Among the 110 participants included, 1 non-autistic adult was excluded from the agency analysis as he never chose 1 of the 3 response keys. Moreover, 1 autistic adult was excluded from the reward analysis, as he ended up with less than 50% valid trials (after removal of anticipations and omissions). Group size, female:male ratio, means and standard deviations of age are reported in Table 1 for included participants.

**Table 1:** Sample description.

Group	Younger children		Older children		Adults	
	n (F:M)	Age mean (SD)	n (F:M)	Age mean (SD)	n (F:M)	Age mean (SD)
Autistic	13 (1:12)	8.5 (1.2)	17 (0:17)	13.0 (2.0)	25 (4:21)	23.2 (4.9)
Non-autistic	17 (6:11)	8.4 (1.1)	14 (5:9)	12.7 (1.8)	24 (6:18)	23.5 (4.9)

### Statistical approach

To analyse data, we adopted a model comparison approach, which allows for the selection of the most plausible statistical model given the data and a set of candidate models [55]. Through separated sets of model comparisons, different research hypotheses were specified as statistical models, and their statistical evidence was evaluated. For each dependent variable, a set of models were compared through the Akaike Information Criteria Weights (AICcWt) (i.e., the probability of each model, given the data and the set of considered models), using the ’AICcmodavg’ [56] R package. Then, likelihood ratio tests were used to compare the different models and test the effects predicted by the most plausible model. In addition, the proportion of variance explained by the most plausible statistical model was calculated [57].

All analyses have been run in R, version 4.0.2 [58].

### Variables

In our analyses, we considered 2 dependent variables. Choices indicate which response key was selected in each trial. RT (Reaction Time) is a continuous non-normally distributed variable that measures the time from the appearance of the central stimulus to the response. For all analyses, data were used in the long form, with as many lines as many valid trials were included for each participant. An exploratory approach was elected to test different potential hypotheses linking each dependent variable to the predictors of interest. Button probability is a within-subjects 3-level categorical factor that indicates the probability of each response button to deliver the effect (low, medium, high). Group is a between-subjects 2-level categorical factor that indicates participants’ group membership (autistic or non-autistic). Age group is a between-subjects 3-level categorical factor that indicates participants’ age (younger children, older children, adults).

*Choices*. Multinomial logistic models were used to identify associations between explanatory factors (group, age group) and participants’ choices among the 3 response options. To this end, we used the *‘gam’* function from the *‘mgcv’* R package [59]. The response option with low (10%) probability of delivering the effect was held as a reference category. Autistic group and younger children were held as reference categories for the group and age group factors, respectively. As such, the multinomial logistic model makes two logistic regressions, comparing the baseline with each of the other 2 categories (e.g., if the baseline is set at "low" it makes one logistic with "low" and "medium" and one with "low" and "high"). The probability that the participant’s response falls into each of the categories is thus estimated. We compared the models that follow.

- **m0** specified the hypothesis of no difference due to the independent variables and only accounted for individual variability
- **m1** specified the hypothesis of a group effect
- **m2** specified the hypothesis of additive group and age group effects
- **m3** specified the hypothesis of a two-way interaction effect between group and age group

*Reaction time (RT)*. Generalised mixed-effects models were employed to analyse the associations between explanatory factors (button probability, group, age group) and RT, thus specifying its gamma distribution (i.e., positively skewed). To this end, we used the *‘glmer’* function of the *‘lme4’* R package [60]. All models accounted for the random effect of participants (i.e., interpersonal variability). We compared the models that follow.

- **m0** (null model) specified the hypothesis of no difference due to the independent variables and only accounted for individual variability
- **m1** specified the hypothesis of differences due to the probability of each response option to deliver a neutral (agency task) or positive (reward task) effect
- **m2** specified the hypothesis of differences due to the probability of each response option to deliver a neutral (agency task) or positive (reward task) effect, with the additive contribution of group membership
- **m3** specified the hypothesis of differences due to the probability of each response option to deliver a neutral (agency task) or positive (reward task) effect, with the additive contribution of group and age group membership
- **m4** specified the hypothesis of differences due to two-way interaction between the probability of each response option to deliver a neutral (agency task) or positive (reward task) effect and group membership, with the additive contribution of age group membership.

### Data pre-processing

Participants completed n = 11,132 trials in the agency task and n = 11,240 trials in the reward task. Raw data were first cleaned from omissions (i.e., participants’ response not within 2000 ms from stimulus presentation), with 1.79% trials being rejected in agency task and 0.84% trials being rejected in reward task. Afterwards, we applied a filter on Reaction Time (RT). Specifically, we excluded those responses whereby RT was less than 100 ms, being ascribable to anticipations. Filtered responses (agency: 406/10,404 = 3.90%; reward: 768/10,876 = 7.06%) were removed and not further analysed. Final dataset included n = 9,998 observations in the agency task and n = 10,108 observations in the reward task.

## Results

### Descriptive statistics

Descriptive statistics (means and standard deviations) are reported in Table 2.

**Table 2:** Descriptive statistics of choices and RT.

Agency	Percentage of choice (%)	Age group		Younger children			Older children			Adults		
		Probability of neutral effect		Low	Medium	High	Low	Medium	High	Low	Medium	High
		Group	Autistic	38.3 (16.6)	25.7 (11.2)	36.0 (15.4)	35.6 (16.1)	32.3 (12.9)	32.1 (13.1)	35.7 (12.6)	32.4 (11.0)	31.9 (13.1)
			Non-autistic	32.6 (6.2)	33.3 (6.0)	34.1 (8.3)	35.9 (10.6)	32.1 (8.7)	32.0 (8.6)	30.1 (12.1)	35.9 (9.9)	34.0 (13.2)
	RT (ms)	Age group		Younger children			Older children			Adults		
		Probability of neutral effect		Low	Medium	High	Low	Medium	High	Low	Medium	High
		Group	Autistic	736.8 (440.1)	744.1 (434.5)	714.6 (442.4)	846.6 (432.7)	831.2 (465.9)	784.4 (441.5)	730.1 (358.2)	718.3 (372.2)	720.8 (379.5)
			Non-autistic	761.6 (365.0)	793.9 (379.8)	802.7 (409.0)	925.4 (396.5)	956.6 (387.3)	910.5 (385.4)	891.2 (325.1)	891.2 (335.9)	896.7 (332.9)
Reward	Percentage of choice (%)	Age group		Younger children			Older children			Adults		
		Probability of positive effect		Low	Medium	High	Low	Medium	High	Low	Medium	High
		Group	Autistic	32.3 (17.3)	27.8 (18.5)	39.9 (16.3)	27.1 (12.1)	28.6 (13.7)	44.3 (18.6)	32.0 (14.7)	29.3 (12.6)	38.7 (18.6)
			Non-autistic	33.6 (16.2)	30.1 (11.4)	36.3 (17.6)	29.8 (11.5)	36.4 (11.0)	33.8 (9.0)	32.6 (12.5)	35.1 (9.5)	32.3 (11.5)
	RT (ms)	Age group		Younger children			Older children			Adults		
		Probability of positive effect		Low	Medium	High	Low	Medium	High	Low	Medium	High
		Group	Autistic	618.7 (397.2)	623.6 (426.2)	581.4 (403.7)	628.6 (412.2)	663.6 (420.6)	658.2 (434.5)	608.8 (350.9)	593.0 (365.0)	577.9 (345.9)
			Non-autistic	728.9 (352.6)	739.3 (382.9)	688.0 (370.8)	851.6 (373.3)	853.2 (409.2)	842.7 (398.7)	760.5 (360.1)	749.4 (358.6)	827.8 (395.0)

Mean (standard deviation).

### Model comparisons

#### Agency

**Fig 2:**
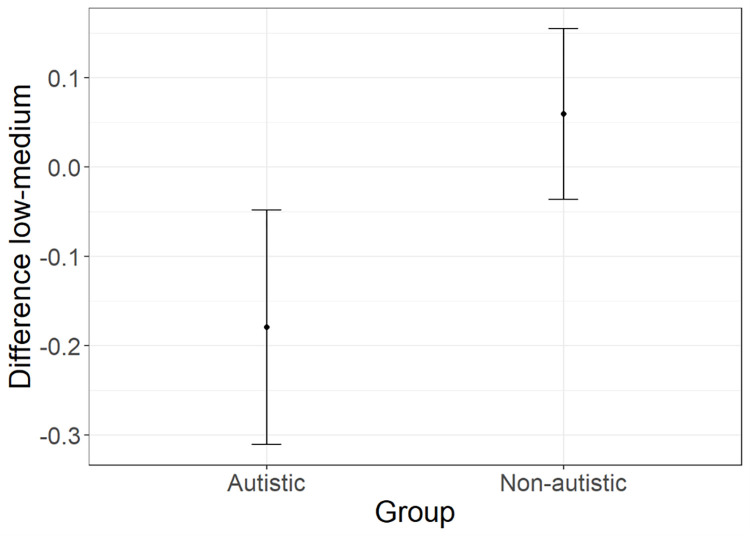
Group effect predicted by m1. Relative choice of the response with medium—vs. low—probability of delivering a neutral vs. no effect, with whiskers representing 95% confidence intervals.

*Choices*. According to AIC weights (AICWt_m0 = .22; AICWt_m1 = **.29**; AICWt_m2 = .25; AICWt_m3 = .24), the most plausible model is **m1** (deviance explained = 5.11%), which revealed a significant group effect in the difference between participants’ choices of the response option with low or medium probability of delivering a neutral vs. no effect (z = 2.24; p = .02). A graphical representation of this effect is displayed in Fig 2. The non-autistic group equally selected the low- and medium-probability response option (confidence interval includes 0 value). On the other hand, the likelihood that the autistic group would select the medium probability option is reduced compared to the likelihood of selecting the low-probability one.

**Fig 3:**
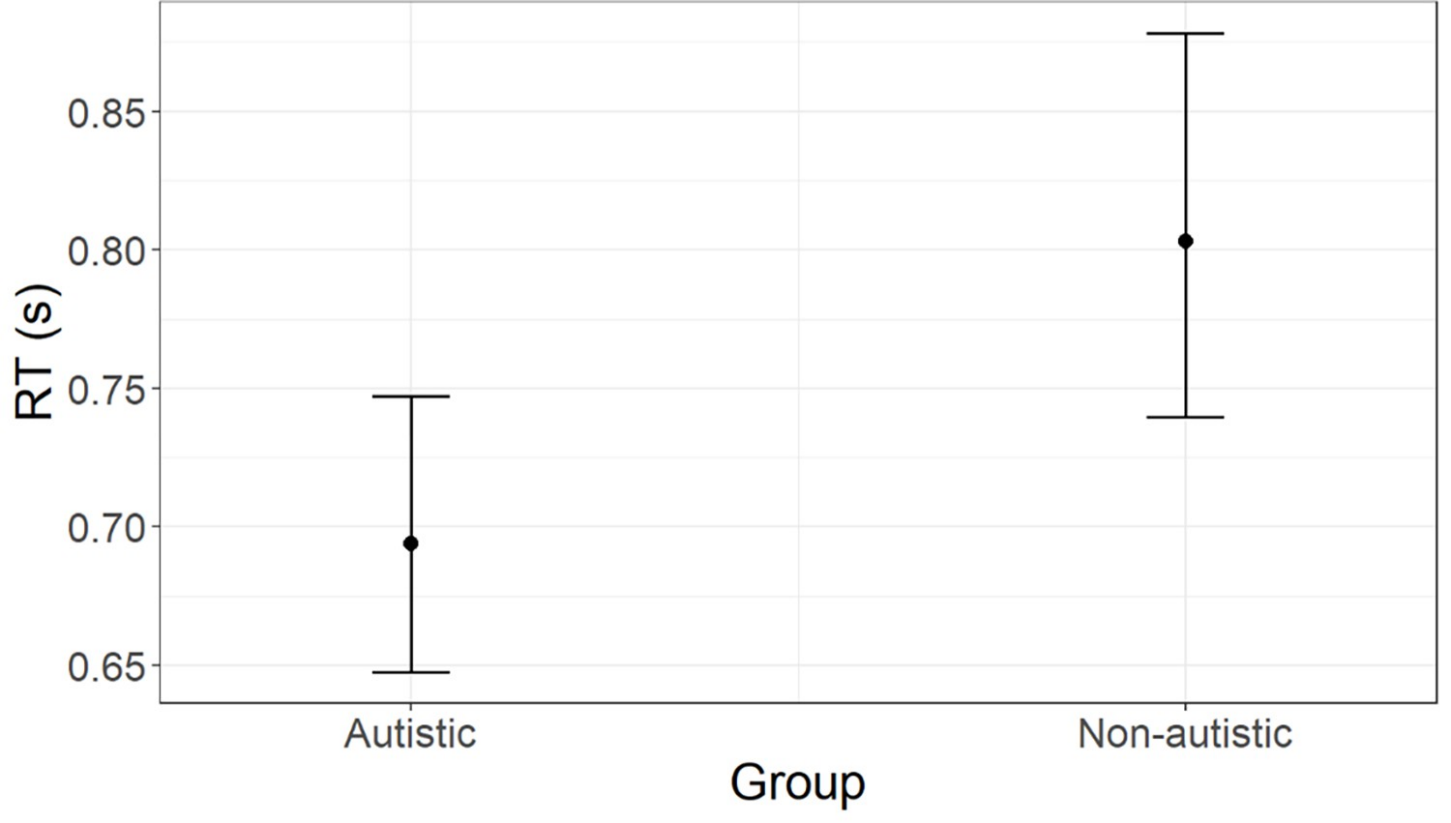
Group effect on RT (agency task), as predicted by m2. Estimated marginal means with whiskers representing 95% confidence intervals.

*RT*. According to AIC weights (AICWt_m0 = .22; AICWt_m1 = .05; AICWt_m2 = **.46**; AICWt_m3 = .23; AICWt_m4 = .04), the most plausible model is **m2** (χ^2^ = 6.66; p = .01) and reveals a significant effect of group (t = -2.62; p = .009). As visualised in Fig 3, the autistic group can be expected to show an overall shorter RT. Predicted RT [95% CI] for the autistic group is 0.69 s [0.75, 0.65], whereas for the non-autistic group is 0.80 s [0.88, 0.74]. Marginal R^2^ = .04, which indicates the ratio of variance explained by fixed effects and the total variance (excluding the contribution of individual variability). Results from the Levene test on the homogeneity of variance lead to the rejection of the null hypothesis that the variances are equal in the autistic and non-autistic groups (F = 61.75; p < .001). Autistic people show more variable RT (see standard deviations reported in Table 2).

#### Reward

**Fig 4:**
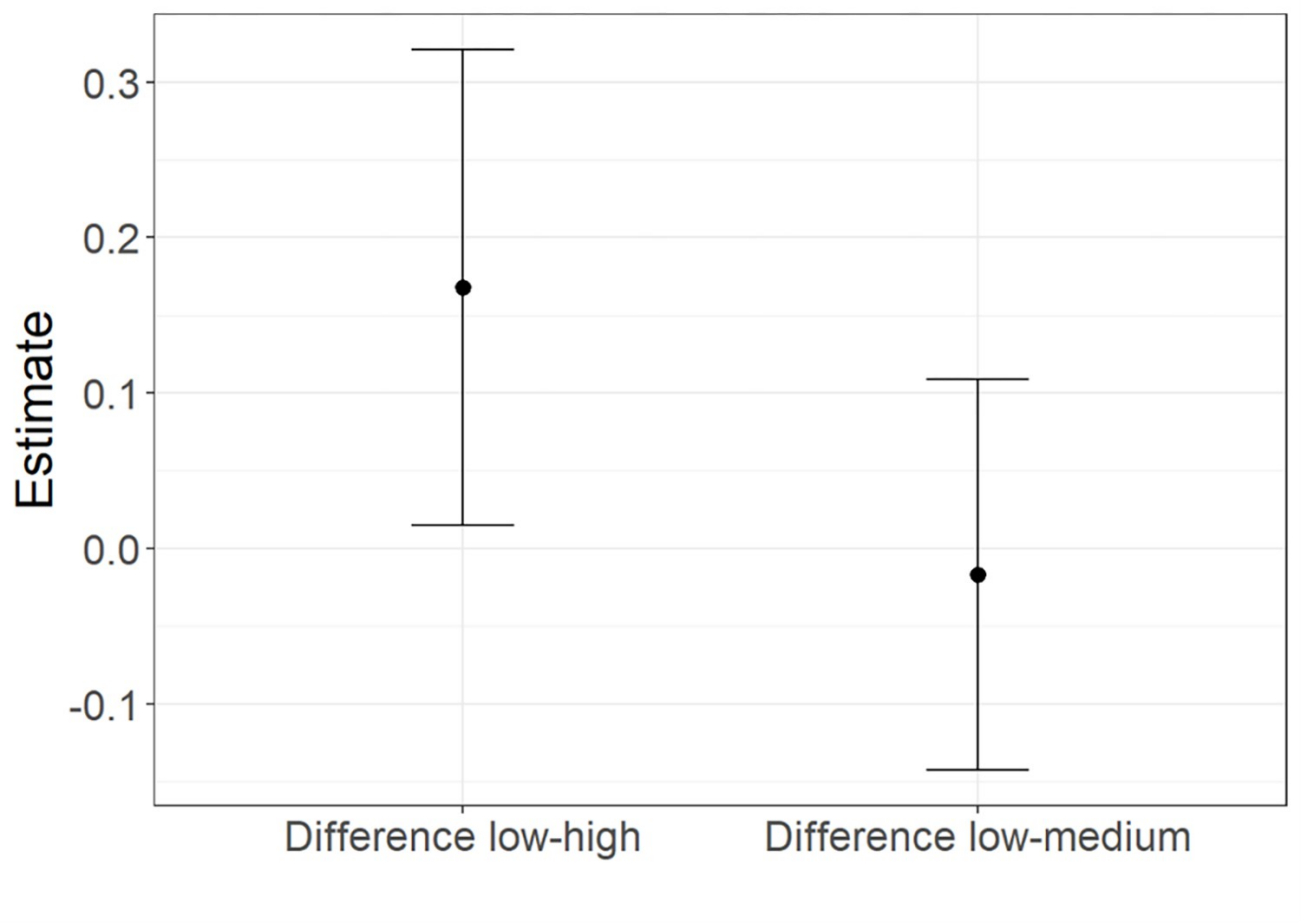
Button probability effect predicted by m0. Relative choice of the response with low vs high and low vs medium probability of delivering a positive vs. neutral effect, with whiskers representing 95% confidence intervals.

*Choices*. According to AIC weights (AICWt_m0 = .26; AICWt_m1 = .26; AICWt_m2 = **.29**; AICWt_m3 = .20), the most plausible model is **m2** (deviance explained = 7.92%), whereby no effects are significant. The second most plausible models are **m0** and **m1**, which compared to m2 have similar probability of being the best models and explain the same amount of deviance (AICWt_m0_m1 = .26; deviance explained = 7.91%). Both m0 and m1 reveal a significant difference in participants’ choices of response options with low or high probability of delivering a positive vs. neutral effect (for m0, z = 2.15; p = 0.03; for m1, z = 2.72; p = .006). A graphical representation of this effect is displayed in Fig 4. The likelihood that participants would choose the response with high probability of a positive vs neutral effect is greater than the probability of choosing the response with low probability of a positive effect. No difference (confidence interval including 0 value) can be found between the probability of choosing the response with medium or low probability of a positive effect.

**Fig 5:**
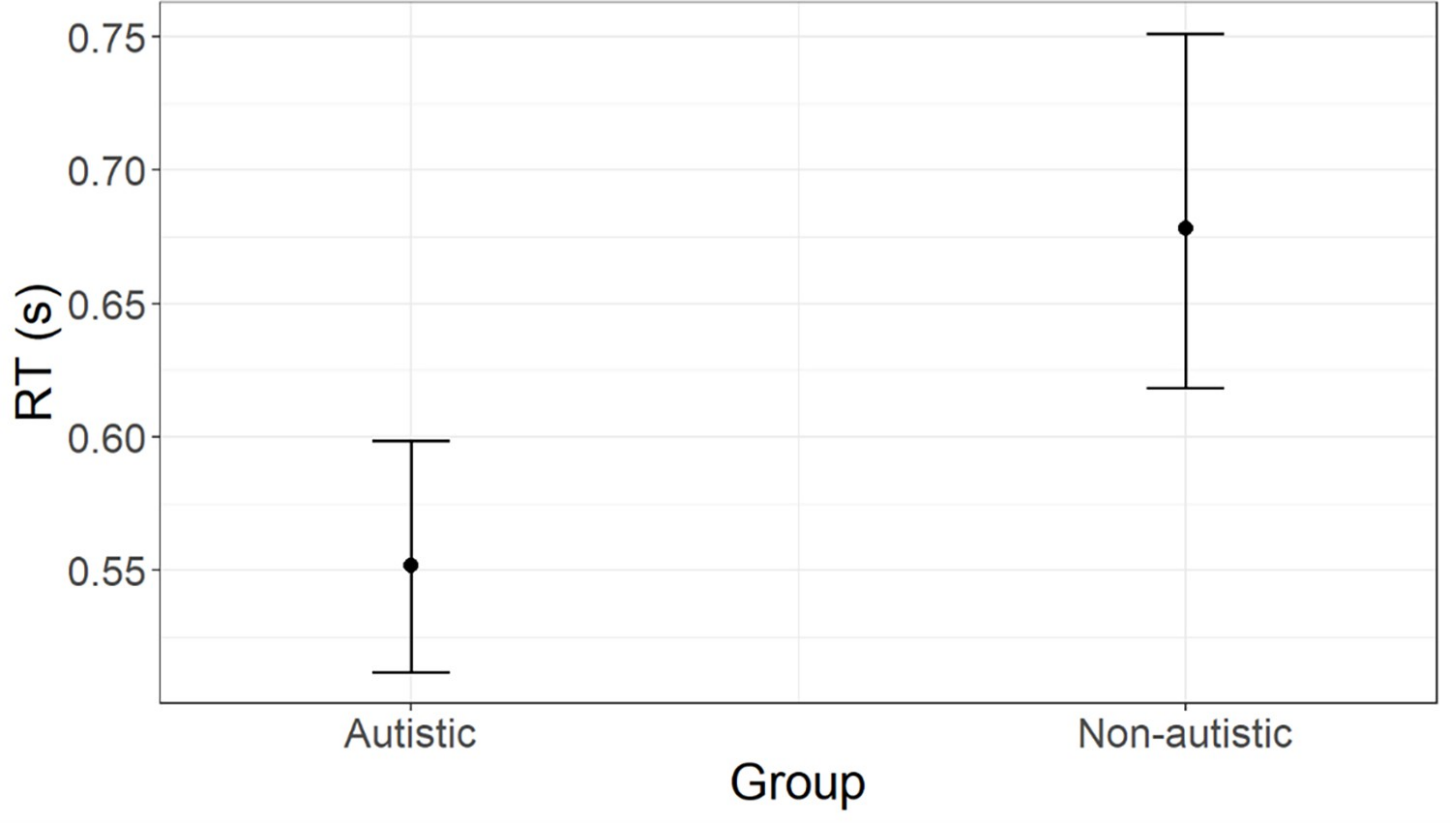
Group difference in RT (reward task), as predicted by model m2. Estimated marginal means with whiskers representing 95% confidence intervals.

*RT*. According to AIC weights (AICWt_m0 = .02; AICWt_m1 = .01; AICWt_m2 = .71; AICWt_m3 = .15; AICWt_m4 = .11), the best model is **m2** (χ^2^ = 10.54; p = .001), which predicts a significant effect of group (t = -3.33; p < .001). As depicted in Fig 5, the autistic group can be expected to show an overall shorter RT. Predicted RT [95% CI] for the autistic group is 0.55 s [0.60, 0.51], whereas for the non-autistic group is 0.68 s [0.75, 0.62]. Marginal R^2^ = .08, which indicates the ratio of variance explained by fixed effects and the total variance (excluding the contribution of individual variability). Results from the Levene test on the homogeneity of variance lead to the rejection of the null hypothesis that the variances are equal in the autistic and non-autistic groups (F = 6.36; p = .01). Autistic people show more variable RT (see standard deviations reported in Table 2).

### Exploratory correlations with AQ

**Fig 6:**
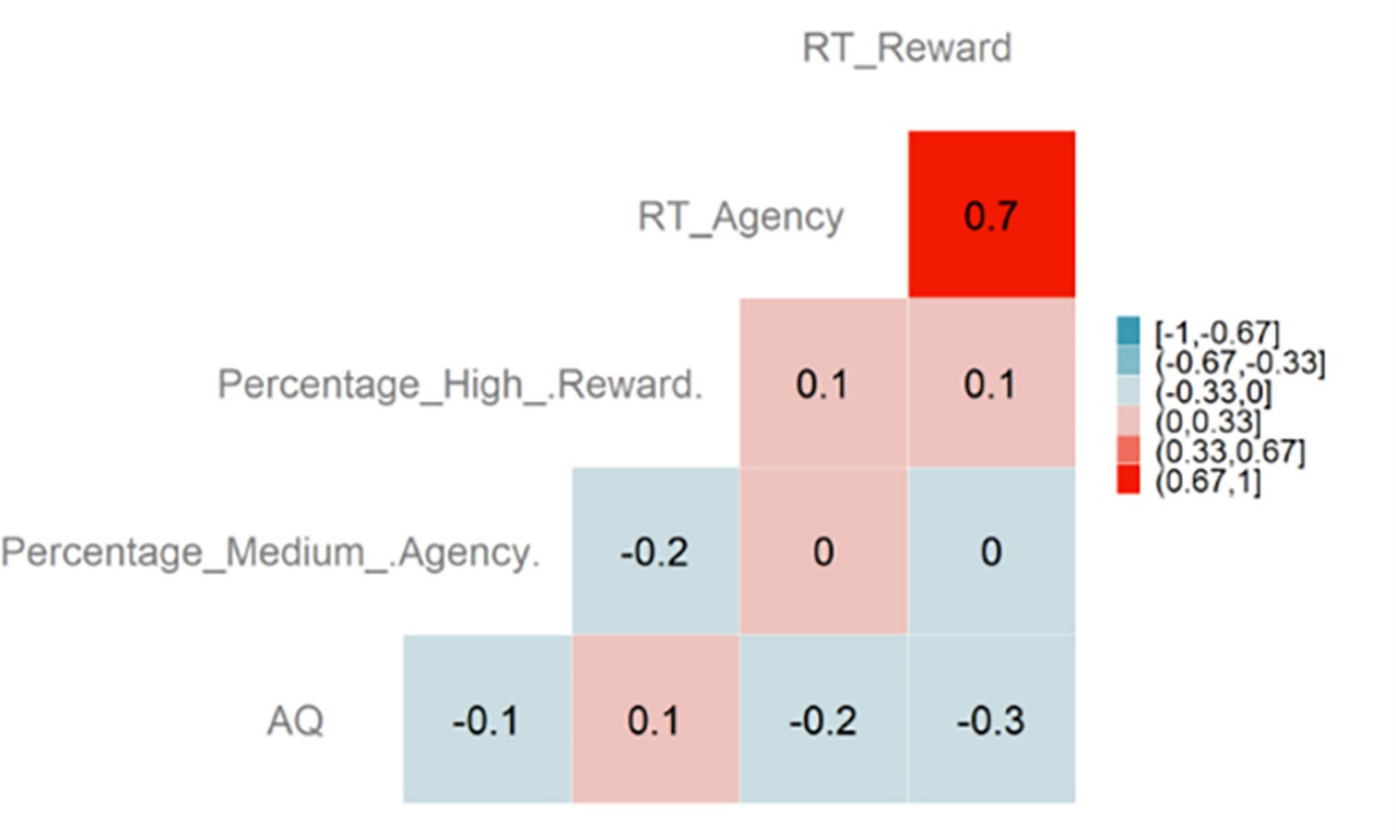
Correlations with autistic traits. n = 42.

In order to assess possible relationships between the results obtained from the model comparison and individual differences, we decided a posteriori to conduct a correlation analysis of certain indices of interest and the autistic traits measured through the AQ questionnaire. Across both the non-autistic and autistic groups, the AQ was compiled by participants above 16 years of age (n = 42). The matrix in Fig 6 shows the pairwise correlations between:

- AQ: scores from the self-reported questionnaire on autistic traits among adults (min = 5; max = 35). Higher values indicate more pronounced autistic traits.
- Percentage_Medium_Agency: percentage of choice for the response option with medium probability of delivering a neutral vs. no effect. Percentages for each participant are calculated over their total number of valid trials.
- Percentage_High_Reward: percentage of choice for the response option with high probability of delivering a positive vs. neutral effect. Percentages for each participant are calculated over their total number of valid trials.
- RT_Agency: average RT in the agency task.
- RT_Reward: average RT in the reward task.

Apart from the RTs that are highly correlated across the two tasks, correlations are overall low or null. Negative correlations between AQ and RTs are appreciated, indicating that individuals with more pronounced autistic traits show slightly shorter RTs.

## Discussion

Our ability to perform actions and make choices is fundamental in our daily interaction with the world of physical and social objects. The link between a given action and its effects on the surrounding environment modifies our behaviour and the underlying cognitive and neural processes, with meaningful effects on our acting, thinking, and learning. Perception of control over the effects of one’s actions (agency) and sensitivity to positive outcomes (reward) increase action frequency and facilitate action planning. These mechanisms undergo developmental changes that can contribute to neurodiverse developmental trajectories. We experimentally investigated the role of agency and reward in driving people’s action selection across the lifespan and in autism. Frequency of choices and reaction times (RT) have been measured while children and adults performed free choices among options with different probability (low, medium, high) of causing an effect. Across two tasks, we separately tested the contribution of agency (i.e., causing a neutral effect compared to no effect at all) and reward (i.e., causing a positive compared to a neutral effect).

In terms of agency, our data showed no preference for participants in choosing response options with a higher probability of giving a neutral versus no effect. In fact, we found no agency effect on the frequencies of choice among the different options, nor on reaction times. Consequently, our results are not in line with the Control-Based Response Selection theory (CBRS) [9] that proposes that the mere action-effectiveness facilitates action selection and speed when people make free-choices among options. To explain the discrepancies between the predictions of the CBRS model [9] and our data we should consider some specific features of our experimental adaptation. While the original study explicitly required participants to perform random response selection [7], we used a cover story designed to engage younger children. Therefore, during the instructions, we told participants to use the response options as if they were candies to feed the animals that appeared on the screen. It is possible that this instruction drove attention towards finding matches between animals and candies, and away from the effects of the selected action. However, this should not be a limitation since the original task, like ours, is designed to elicit mechanisms of implicit agency, which goes beyond conscious intentions and evaluations. It is also possible that such a simple and un-naturalistic task has limited potential in capturing the essence of agency (and driving people’s choices depending on mere action-effectiveness manipulations), which should be further explored in ecological situations where the action-consequence link takes on real relevance for the person.

In terms of reward, we found a general preference for choosing the option with a higher probability of a positive versus neutral effect. However, the effect valence did not affect reaction times, thus failing to facilitate motor preparation and execution in our task. Previous literature offers contradictory findings on whether positive outcomes can actually facilitate actions at the motor level. The CBRS literature found that monetary outcomes of adults’ free choices do not decrease RTs [9]. Other authors found evidence that monetary rewards make actions faster, with reward magnitude being associated with the activation of pre-SMA and SMA brain areas, potentially promoting motor planning prior to action execution [16]. Differently from these previous studies, we used a reward stimulus (smiley) with immediate positive value even for people with a limited concept of the value of money (i.e., very young children and some autistic participants). It is possible that the social characteristics of our reward stimulus involved different mechanisms than those devoted to processing non-social rewards. Indeed, even very simplified stimuli that resemble the global structure of faces activate specific subcortical and cortical routes of the social brain [61]. Interestingly, different types of feedback can have a different motivational effect for people with different ages and developmental trajectories. The literature points out that neurotypical children are differently motivated by social and non-social rewards in different situations [62]. Autism research, on the other hand, presents evidence on possible differences in neural reward circuits [46–48], with reduced motivation from and search for social outcomes [25,63].

As for the autistic population, the study has an exploratory intent. Little is known about how these processes function and develop across typical development, so it is risky to draw definitive conclusions about autism. However, we believe it is of interest for autism research to make some preliminary observations from the data we have analysed, bearing in mind that those should be taken with caution and further studied in future research.

First, we found a group difference in the agency task. While the non-autistic group equally selected the response option regardless of their probability of a neutral vs. no effect, the autistic group seemed to avoid the response option with chance probability (50%) of giving a neutral vs. no effect. Since that response option can be seen as the one with highest level of uncertainty, we can interpret this finding in light of the evidence on the intolerance of uncertainty in autistic individuals [64], who might overestimate the volatility of the environment and be more prone to expect the unexpected [65]. In this sense, autistic participants may have avoided choosing the response key associated with higher uncertainty about the outcome. Intolerance for uncertainty is a potential mechanism underlying the presence of restricted and repetitive behaviours in autism [66,67]. However, among the adults that participated in this study, the frequency of choosing that response option did not correlate with autistic traits.

Second, we did not find any reward-specific differences between non-autistic and autistic groups. Overall participants more frequently selected the option with higher probability of a positive vs. neutral effect. We can interpret this finding as a sign of sensitivity to and motivation from reward. This is not in line with previous literature on reduced motivation from reward in autistic people [47,51,63].

Third, autistic participants, across all age groups and the two tasks, showed shorter and more variable reaction times than non-autistic participants. We found a small negative correlation between autistic traits and RTs, with more pronounced autistic traits being associated with shorter RTs (the analysis was run on data from all participants older than 16, regardless of their formal diagnosis of autism). These findings may indicate the presence of a general propensity for more automatic responses, but also increased variability and therefore uncertainty in the action timing (for a theoretical conceptualisation of predictive processing and volatility in autism, see [68]). Reduced RTs can be interpreted as a marker of less time spent planning the action before its execution and/or less time devoted to online monitoring and control of the ongoing movement [69–71]. In everyday life, people constantly perform actions that require both planning and control. Previous findings suggested that when autistic people perform self-generated actions, their movements can become stereotyped [72]. Stereotypies are involuntary, restricted, and repetitive patterns of movements that limit the resources to learn and practise various, purposeful actions [43,44,73]. Ineffective motor planning seems to be associated with motor stereotypies [42], which are present in autism, other neurodevelopmental conditions and typical development [73]. Notably, motor-related cortical potentials in premotor areas, which anticipate voluntary motor actions are found to be absent before stereotypy onset in typical development [42]. We can speculate that reduced action planning in autism is related to reduced reliance on predictions about the action outcomes [68], potentially contributing to difficulties in learning from the action consequences. In this perspective, sub-optimal predictive processes and action planning could be associated with reduced sensitivity to agency and reward. That is suggested by previous literature but not clearly emerged from the current study. Further research is needed to investigate these aspects in autism, digging into the implications for the individuals’ daily life and well-being.

### Limitations

Our model comparisons suggest that there are in fact some differences in the extent to which participants select response options according to their probability of producing specific effects. Specifically, all groups more frequently selected the response option with higher probability of a positive vs. neutral effect. Moreover, the autistic group seemed to avoid selecting the response option with chance level of producing a neutral or no effect. We also found group differences in overall reaction times, with the autistic group showing shorter RT. These considerations are supported by the information criteria that led to the selection of the most plausible models in explaining our data. However, the models explained a limited amount of variability within the data, as suggested by the deviance explained for the ‘choices’ variable, and the R^2^ for the RT variable, with the most plausible models only explaining from 5 to 8% of variability. Therefore, our results should be interpreted with caution, and future work should investigate additional latent variables that may contribute to the phenomena under study.

A limitation of the current study is sample size, which was determined by the number of families that agreed to participate in the study. While the original CBRS studies on neurotypical adults used a much larger sample [7,9], it is possible that our study was under-powered to detect agency and reward effects that might be actually small in the general population. Given the paucity of prior evidence on children and autistic populations, we were not able to a-priori estimate expected effect sizes and appropriate sample sizes, resulting in a sample size that may be insufficient to reveal further patterns within the data. Relatedly, although participants over the age of 16 filled out the AQ questionnaire, thus providing a measure of the presence of autistic traits, participants were not characterized in terms of cognitive and verbal functioning. The heterogeneity within the autism spectrum along the cognitive and verbal dimensions might have contributed to between-group differences (for example in reaction times) that go beyond autism-specific characteristics. For these reasons, our results should be considered as a first exploratory step towards investigating how agency and reward drive free choices across different developmental trajectories. Further studies with appropriate sample size should delve into the role of individual differences. For instance, the CBRS framework proposed that agency, or reward from control, may explain behaviours such as stereotypy [7]. However, to the best of our knowledge this link has not yet been directly tested in the literature. We chose autism as a condition of interest for this study, as the presence of repetitive behaviours and stereotypies are one of the two diagnostic criteria of the condition. As a future perspective, having a direct measure of the presence of stereotypies in autistic or other populations will help to shed light on the possible relationship between these behaviours and agency mechanisms.

Lastly, as we were not interested in assessing gender differences, our sample is not balanced by participant gender, which reduces its representativeness of the general population.

### Future perspectives

The literature review and discussion of the experimental results open the door to challenging perspectives for future research. During naturalistic interactions with the outside world, a sense of agency and the reward derived from the positive consequences of one’s actions are closely interconnected. Indeed, when an action that the agent perceives as voluntary has a consequence that is interpreted as self-caused and also positive, the two experiences are concomitant. Motivation from agency and reward are therefore frequently bounded together [13,14,17]. To better disentangle these mechanisms, the use of neural measures of agency and reward will offer unique possibilities. Distinct and shared components of neural activity are associated with the sense of agency and sensitivity to reward. For example, preparatory neural activity in motor and premotor areas anticipates voluntary movements and contributes to implicit agency [15] but is also affected by reward [16]. Distinct neural components are instead in charge of processing what happens after the action and its outcomes. While sensory-related activity is associated with the sense of agency in response to expected self-caused outcomes [74,75], neural activity in amygdala and thalamus areas underlie reward consumption [76].

We are far from understanding how these different mechanisms specialise during the course of child development and may be involved in neurodiverse trajectories. Our behavioural task is a first attempt to establish a paradigm suitable for young children and people with limited verbal abilities, which can be integrated with neural measures to shed light on the deep mechanisms underlying motivation from agency and reward. Nevertheless, the extant literature and the present study mainly employed simple and un-naturalistic tasks that might have limited potential in capturing the essence of agency and reward, and their role in driving people’s actions and choices, which should be further explored in ecological situations where the action-consequence link takes on real relevance for the person. Agency is also crucial during interpersonal exchanges, whereby each partner of the interaction influences the behaviour of the other through his or her own verbal and non-verbal initiatives, and thus feels that he or she has an active role in the exchange [77]. Whereas agency research has focused on the use of non-social effects of action, it would be extremely important for future studies to examine the developmental trajectories of agency in social and non-social situations.

Broadening the focus to the processes underlying individuals’ choices and actions, the motivation from agency and reward interacts with higher-level cognitive processes, which we can generally refer to as executive functions. For instance, action planning and control difficulties (that we have found in the autistic sample, as suggested by their reduced RTs) may be associated with reduced ability to inhibit prepotent responses and routine behaviours [70], thus impacting the way people make complex choices among numerous possible alternatives in everyday life situations. Although agency and reward are fundamental mechanisms for learning, it is crucial that our repertoire of actions is flexible, that we are able to reorient our choices when needs change. For this, we must also be able to take actions over whose consequences we do not have absolute control, or which have uncertain and potentially negative effects. Future research should investigate the interplay between agency, reward, and executive functions such as inhibition and cognitive flexibility, to understand not only how people learn from the effects of their actions, but also how this learning is open to change and constant cognitive monitoring and control.

Interesting prospects also open up for autism research and interventions. Leveraging both agency and reward may be crucial for enhancing the action-outcome binding that is pivotal for a person to learn from their own actions, while also fostering self-determination. From early in life, the child having the control over the environment is a key element that mediates increased attention and reduced repetitive and stereotyped behaviours [78]. Providing control over sensory changes to children may create better conditions for learning. On the other hand, there are different ways of using rewards to facilitate the child’s learning. Behavioural interventions for children on the autism spectrum often employ “artificial” rewards (e.g., food or tokens) to reinforce behaviours during adult-determined activities. Instead, recently operationalised, evidence-based interventions that follow under the umbrella category of Naturalistic Developmental Behavioural Interventions (NDBIs), recommend reinforcing the child’s behaviours with “natural” consequences [79]. Using the example of language learning, when a child says “train” to express their intention to play with a toy train, the adult can reinforce this behaviour with affective facial and vocal expressions (smiling and saying “yes, a train!”), and body language (pointing to the train). Those naturalistic reinforcements that are related to the activity and foster social engagement should be preferred to artificial rewards (e.g., giving chocolate or tokens to reinforce target behaviours). Importantly, the adult should “follow the child’s lead”, creating learning opportunities from the child’s initiative, interests, and preferred activities [79].

The investigation of the intra-individual and neuropsychological mechanisms that shape the way autistic individuals select actions and make choices does not neglect that they are situated and emergent from social, cultural, educational, and political contexts that shape the contours of “ability” and “disability”. Removing barriers to volition and self-determination is crucial when offering support and learning opportunities to individuals.

## Conclusions

The Control-Based Response Selection theory (CBRS) proposes that the mere action-effectiveness facilitates action selection and speed when people make free choices among options. The model hypothesises that motivation by this sense of control often disregards the valence of the effect of one’s action and may be atypical in people who exhibit repetitive behaviours such as stereotypies, which are often present in autism. Our results did not show this agency effect in facilitating the free choices of non-autistic or autistic children and adults. We did, however, find such facilitation for options with a higher probability of resulting in positive effects (reward). In the group of participants with autism, we found signs of reduced tolerance of uncertainty (more infrequent choice of options with a more uncertain outcome), shorter response times (interpretable as a marker of less planning and control of the choice) and greater variability in action processes. Further studies are needed to investigate the neurodiverse mechanisms and individual differences of choice-making in autism, to understand the origins and functions of repetitive behaviours, facilitate flexibility and leveraging self-determination.
