# Supplementary material for: Agency and reward across development and in autism: A free-choice paradigm
Source: PLoS One. 2023 Apr 12;18(4):e0284407. doi: 10.1371/journal.pone.0284407 (PMC10096291; doi:10.1371/journal.pone.0284407)
Supplement: Peer review history

# Peer review: Decision Letter 0

**DOI:** 10.1371/journal.pone.0284407.r001

**Authors:** Yukiori Goto

14 Mar 2023

PONE-D-23-02212Agency and reward across development and in autism: a free-choice paradigmPLOS ONE

Dear Dr. Farroni,

Thank you for submitting your manuscript to PLOS ONE. After careful consideration, we feel that it has merit but does not fully meet PLOS ONE’s publication criteria as it currently stands. Therefore, we invite you to submit a revised version of the manuscript that addresses the points raised during the review process.

Please submit your revised manuscript by Apr 28 2023 11:59PM. If you will need more time than this to complete your revisions, please reply to this message or contact the journal office at plosone@plos.org. When you're ready to submit your revision, log on to https://www.editorialmanager.com/pone/ and select the 'Submissions Needing Revision' folder to locate your manuscript file.

Please include the following items when submitting your revised manuscript:

- A rebuttal letter that responds to each point raised by the academic editor and reviewer(s). You should upload this letter as a separate file labeled 'Response to Reviewers'.
- A marked-up copy of your manuscript that highlights changes made to the original version. You should upload this as a separate file labeled 'Revised Manuscript with Track Changes'.
- An unmarked version of your revised paper without tracked changes. You should upload this as a separate file labeled 'Manuscript'.

If you would like to make changes to your financial disclosure, please include your updated statement in your cover letter. Guidelines for resubmitting your figure files are available below the reviewer comments at the end of this letter.

If applicable, we recommend that you deposit your laboratory protocols in protocols.io to enhance the reproducibility of your results. Protocols.io assigns your protocol its own identifier (DOI) so that it can be cited independently in the future. For instructions see: https://journals.plos.org/plosone/s/submission-guidelines#loc-laboratory-protocols. Additionally, PLOS ONE offers an option for publishing peer-reviewed Lab Protocol articles, which describe protocols hosted on protocols.io. Read more information on sharing protocols at https://plos.org/protocols?utm_medium=editorial-email&utm_source=authorletters&utm_campaign=protocols.

We look forward to receiving your revised manuscript.

Kind regards,

Yukiori Goto, Ph.D.

Academic Editor

PLOS ONE

Journal Requirements:

When submitting your revision, we need you to address these additional requirements.

1. Please ensure that your manuscript meets PLOS ONE's style requirements, including those for file naming. The PLOS ONE style templates can be found at 

https://journals.plos.org/plosone/s/file?id=wjVg/PLOSOne_formatting_sample_main_body.pdf and 

https://journals.plos.org/plosone/s/file?id=ba62/PLOSOne_formatting_sample_title_authors_affiliations.pdf

2. We note that the grant information you provided in the ‘Funding Information’ and ‘Financial Disclosure’ sections do not match. 

When you resubmit, please ensure that you provide the correct grant numbers for the awards you received for your study in the ‘Funding Information’ section.

3. We note that you have stated that you will provide repository information for your data at acceptance. Should your manuscript be accepted for publication, we will hold it until you provide the relevant accession numbers or DOIs necessary to access your data. If you wish to make changes to your Data Availability statement, please describe these changes in your cover letter and we will update your Data Availability statement to reflect the information you provide.

4. Please upload a new copy of Figure 6 as the detail is not clear. Please follow the link for more information: " ext-link-type="uri" xlink:type="simple">https://blogs.plos.org/plos/2019/06/looking-good-tips-for-creating-your-plos-figures-graphics/" " ext-link-type="uri" xlink:type="simple">https://blogs.plos.org/plos/2019/06/looking-good-tips-for-creating-your-plos-figures-graphics/"

Additional Editor Comments:

I am sorry for taking time to complete review process. The reviewers have raised several concerns. Please revise the manuscript by addressing these issues.

[Note: HTML markup is below. Please do not edit.]

Reviewers' comments:

Reviewer's Responses to Questions

**Comments to the Author**

1. Is the manuscript technically sound, and do the data support the conclusions?

The manuscript must describe a technically sound piece of scientific research with data that supports the conclusions. Experiments must have been conducted rigorously, with appropriate controls, replication, and sample sizes. The conclusions must be drawn appropriately based on the data presented. 

Reviewer #1: Partly

Reviewer #2: Yes

**********

2. Has the statistical analysis been performed appropriately and rigorously? 

Reviewer #1: Yes

Reviewer #2: Yes

**********

3. Have the authors made all data underlying the findings in their manuscript fully available?

The PLOS Data policy requires authors to make all data underlying the findings described in their manuscript fully available without restriction, with rare exception (please refer to the Data Availability Statement in the manuscript PDF file). The data should be provided as part of the manuscript or its supporting information, or deposited to a public repository. For example, in addition to summary statistics, the data points behind means, medians and variance measures should be available. If there are restrictions on publicly sharing data—e.g. participant privacy or use of data from a third party—those must be specified.

Reviewer #1: Yes

Reviewer #2: Yes

**********

4. Is the manuscript presented in an intelligible fashion and written in standard English?

PLOS ONE does not copyedit accepted manuscripts, so the language in submitted articles must be clear, correct, and unambiguous. Any typographical or grammatical errors should be corrected at revision, so please note any specific errors here.

Reviewer #1: Yes

Reviewer #2: Yes

**********

5. Review Comments to the Author

Please use the space provided to explain your answers to the questions above. You may also include additional comments for the author, including concerns about dual publication, research ethics, or publication ethics. (Please upload your review as an attachment if it exceeds 20,000 characters)

Reviewer #1: This study reports that autistic subjects exhibited reduced tolerance to uncertainty in the agency task than typically developing subjects. The finding may have a significant merit for publication. The data analysis also appears to be quite adequate, except one issue that I would have a concern. Participants were grouped based on ages, younger children, older, children, and adults. However, what was the rationale behind separating participants into these groups of the specific ages? Were there such significant differences between the age of 10 and 11 years old as well as between 16 and 17 years old that enable to segregate participants into groups? To me, ages are rather continuous, so that this variable also has to be treated as continuous in statistical analysis rather than the way of analysis currently employed.

Minor concern: I am not sure if this is because of the format of the journal, but aim and hypotheses are not materials and methods at all, so these descriptions have to be rather integrated in the introduction.

Reviewer #2: the manuscript "Agency and reward across development and in autism: a free-choice paradigm" is well-written and provides data and script. Would be neat to also provide the material, as this would facilitate replications (combining your task with eye-tracking for example).

I have only a few issues.

I was surprised about the introduction not linking agency to ToM. Agency and intentional binding is seen as a prerequisite for ToM. It was new to me to link it to stereotypes (I am more familiar with stereotypes being a mean to express feelings).

Since you did neither know about the participants repetitive movements / behaviours nor their social cognition, it will not matter. There is also no information about co-morbidities or IQ. You do explain why, however, you also describe that the rational for using a non-verbal task is to include children but also those that do not poses sufficient verbal skills as adults (which often means low IQ). You do find differences in choice pattern (and RT) but I do not think they are due to differences in cognitive ability. Still, it would have been nice to know a bit more about your sample, not just age and gender.

Notably, the RT in the reward task are lower than in the agency task. Here a correlation would be interesting. Is there a higher correlation in the non-autistic group than in the autistic group between RT_agency and RT_reward.

The rational for that is smth we see in ASD and SCZ, there is more noise within the ASD and SCZ group, meaning lower correlation across tasks. This would (see below) support the uncertainty and volatility interpretation, i.e. they are less consistent. Indeed, reporting the SD per group might be informative, I expect the autistic group to be more variable (as Table 2 indicates) - please report the statistical test (if it would be two groups it would be Levene)

It is an interesting but not surprising finding that persons with ASD least choose the condition where they can least predict the outcome, i.e. the 50% condition. If you also find more "noisy" responding (use in a GLM the SD per participant as outcome and group (ASD vs non-ASD) and age group as predictor) and a lower correlation between RT_agency and RT_reward (again using longformat and hence calculate Pearson's r per ID and then compare), you have three independent indications of the ASD group differing from the non-ASD group that align with predictive coding ideas of ASD.

Since you provided the data I had a quick look at the elephant in the room, namely whether the difference in RT can be explained by the gender differences. Your ASD sample has mostly males whereas your control group is more even, and not as skewed in the gender distribution as the ASD sample is. In a model with RT as outcome variable and gender as predictor I got a significant effect (can't nest within group as one group had no females, e.g. older children).

Table 1 also has for non-autistic adults n=24 (6:8) but 6+8 = 14, is it 6:18 which would be a good match to the autistic group or 16:8?

I would be interested to see a model with gender and whether it wins over model 1 and 2.

Finally, I missed a conclusion. You have a lengthy section on future perspectives. This is fine but the reader does not know what the take home message is.

**********

6. PLOS authors have the option to publish the peer review history of their article (what does this mean?). If published, this will include your full peer review and any attached files.

If you choose “no”, your identity will remain anonymous but your review may still be made public.

**Do you want your identity to be public for this peer review?** For information about this choice, including consent withdrawal, please see our Privacy Policy.

Reviewer #1: No

Reviewer #2: No

**********

[NOTE: If reviewer comments were submitted as an attachment file, they will be attached to this email and accessible via the submission site. Please log into your account, locate the manuscript record, and check for the action link "View Attachments". If this link does not appear, there are no attachment files.]

While revising your submission, please upload your figure files to the Preflight Analysis and Conversion Engine (PACE) digital diagnostic tool, https://pacev2.apexcovantage.com/. PACE helps ensure that figures meet PLOS requirements. To use PACE, you must first register as a user. Registration is free. Then, login and navigate to the UPLOAD tab, where you will find detailed instructions on how to use the tool. If you encounter any issues or have any questions when using PACE, please email PLOS at figures@plos.org. Please note that Supporting Information files do not need this step.

# Author comment: Author response to Decision Letter 0

**DOI:** 10.1371/journal.pone.0284407.r002

24 Mar 2023

PONE-D-23-02212

Agency and reward across development and in autism: a free-choice paradigm

PLOS ONE

24th March 2023

Response to Reviewers

R: (Reviewer’s comment)

A: (Answer)

Review Comments to the Author

Reviewer #1: This study reports that autistic subjects exhibited reduced tolerance to uncertainty in the agency task than typically developing subjects. The finding may have a significant merit for publication. The data analysis also appears to be quite adequate, except one issue that I would have a concern.

R: Participants were grouped based on ages, younger children, older, children, and adults. However, what was the rationale behind separating participants into these groups of the specific ages? Were there such significant differences between the age of 10 and 11 years old as well as between 16 and 17 years old that enable to segregate participants into groups? To me, ages are rather continuous, so that this variable also has to be treated as continuous in statistical analysis rather than the way of analysis currently employed.

A: We thank the reviewer for rising this concern. Many neuropsychological and motor processes undergo a non-linear and non-monotonic development, which frequently results in U-shaped learning (see here for reference: https://www.frontiersin.org/articles/10.3389/fpsyg.2013.00301/full). This is why age bands based on developmental stages can be particularly useful in developmental research. The 6-10 band represents school age. 11-16 embraces pre- and early-adolescence. From 17 on we include late adolescence and young adulthood. For instance, RT are related to cognitive and motor planning skills that improve during the school age and then decrease in mid-adolescence, when there is a crucial phase in proliferation and pruning of synapses. As some authors found (https://acamh.onlinelibrary.wiley.com/doi/full/10.1111/j.1469-7610.2006.01611.x): “A 10–20% increase in reaction time occurred at the onset of puberty in the 10–11-year-old group of girls and in the 11–12-year-old group of boys, compared to the previous year group of each sex (age 9–10 and 10–11 in girls and boys, respectively). The results suggest that there is a dip in performance on this kind of task at the onset of puberty. After puberty, from age 13–14, performance improved until it returned to the pre-pubescent level by the age of about 16–17 years”.

We have now expanded on the rational for the 3 age groups in the manuscript:

“We have selected these age ranges in the light of the extant knowledge of discontinuous cognitive development between school age, adolescence, and adulthood. Specifically, between the ages of 10-11 and 16-17, there is an increase in reaction times to certain cognitive tasks, commonly attributed to the processes of synaptic proliferation and pruning that occur at puberty and adolescence (53).”

Please see below for a visualisation of RT distribution and RT-age (continuous) regression lines by age group (1 stands for younger children, 2 for older children, 3 for adults) that go in the direction predicted by the aforementioned previous literature (despite the age_group effect not being significant in our models).

Back to the reviewer’s suggestion, using age as continuous would result in estimating the effect of age on a year-to-year basis. We agree that in theory this is a more informative approach, however without a large sample the possibility of estimating the age effect so finely is limited. Following the reviewer’s input, we’ve run the models using age in the continuous format. In this way, some of the models fail to converge, which is probably related to the insufficient data to model the effect of age as continuous.

R: Minor concern: I am not sure if this is because of the format of the journal, but aim and hypotheses are not materials and methods at all, so these descriptions have to be rather integrated in the introduction.

A: We thank the reviewer for this suggestion, and we moved the aim and hypotheses section accordingly.

Reviewer #2: the manuscript "Agency and reward across development and in autism: a free-choice paradigm" is well-written and provides data and script. Would be neat to also provide the material, as this would facilitate replications (combining your task with eye-tracking for example).

A: We thank the reviewer for this nice suggestion and great idea. We have now set the Labvanced experiment as open access design so that the template can be accessed and used by anyone on the Labvaced platform. The link is reported in the manuscript. https://www.labvanced.com/page/library/29586

R: I have only a few issues. I was surprised about the introduction not linking agency to ToM. Agency and intentional binding is seen as a prerequisite for ToM. It was new to me to link it to stereotypes (I am more familiar with stereotypes being a mean to express feelings). Since you did neither know about the participants repetitive movements / behaviours nor their social cognition, it will not matter.

A: We thank the reviewer for giving us the opportunity to clarify this aspect. The interest in the link between agency and repetitive behaviours is raised by the authors of the theoretical model of reference (control based response selection framework). As Karsh Eitam (2015) stated, “this ‘reward from control’ may explain everyday addictions such as prolonged engagement in arcade games and pathological behaviors, such as stereotypy.” However, to the best of our knowledge this link has not yet been tested in the literature. Instead, the link between reward and stereotypies, often conceptualised as self-stimulation, is more studied. We chose autism as a condition of interest for this study, as the presence of repetitive behaviours and stereotypies are one of the two diagnostic macro-categories of the disorder. We agree that it would have been interesting to have a direct measure of the presence of stereotypies in our autistic participants. We now better discuss it as a limitation and future perspective for further studies.

R: There is also no information about co-morbidities or IQ. You do explain why, however, you also describe that the rational for using a non-verbal task is to include children but also those that do not poses sufficient verbal skills as adults (which often means low IQ). You do find differences in choice pattern (and RT) but I do not think they are due to differences in cognitive ability. Still, it would have been nice to know a bit more about your sample, not just age and gender.

A: We agree with the reviewer that having a better characterisation of the sample would have been informative. Unfortunately, for privacy reasons we could not access the complete medical records and diagnostic information of the participants. In any case, the heterogeneity of the population on the autism spectrum is such that a much larger sample would be needed to investigate the role of individual differences. Fundamental questions remain unanswered and deserve further large-scale studies.

R: Notably, the RT in the reward task are lower than in the agency task. Here a correlation would be interesting. Is there a higher correlation in the non-autistic group than in the autistic group between RT_agency and RT_reward. The rational for that is smth we see in ASD and SCZ, there is more noise within the ASD and SCZ group, meaning lower correlation across tasks. This would (see below) support the uncertainty and volatility interpretation, i.e. they are less consistent. (..run..) correlation between RT_agency and RT_reward (using longformat and hence calculate Pearson's r per ID and then compare).

A: We thank the reviewer for giving us the opportunity to further discuss this interesting aspect. As we disclose in the Procedure, “All participants firstly performed the agency task, and then the reward task, to avoid carryover effects (i.e., a potential reduction of the value of the neutral effect after receiving a positive effect in the previous block of trials).” Therefore, we cannot distinguish whether the different reaction times in the two tasks are due to the fact that the reward effect is greater (in terms of facilitating planning and execution of the choice) or whether it is a general learning effect of the task. As suggested, we did check whether the correlation between RT_agency and RT_reward is different in the two groups. Spearman’s correlations reveal very close indices: rautistic = 0.72 (n=54); rnon-autistic= 0.73 (n=54). We used Spearman’s rather than Pearson’s correlation as RT is non-normally distributed.

R: Indeed, reporting the SD per group might be informative, I expect the autistic group to be more variable (as Table 2 indicates) - please report the statistical test (if it would be two groups it would be Levene)

A: We are grateful to the reviewer for this suggestion. We have run the Levene test on RT_agency (F=61.75; p.001) and RT_reward (F=6.36; p=.01) by group and reported the results in the relevant section of the manuscript (results and discussion). This evidence indeed suggests that autistic participants have more variable RTs.

R: It is an interesting but not surprising finding that persons with ASD least choose the condition where they can least predict the outcome, i.e. the 50% condition. If you also find more "noisy" responding (use in a GLM the SD per participant as outcome and group (ASD vs non-ASD) and age group as predictor) ….. and you have three independent indications of the ASD group differing from the non-ASD group that align with predictive coding ideas of ASD.

A: We thank the reviewer for the insights and practical suggestions. We would first like to clarify how our choice variable is calculated. This is the probability that each participant chooses each of the 3 response keys. As such, we rely on the individual observations (choices) (namely, the dataset in the long format with one row per trial) to calculate this probability. The resulting probability variable, on which the statistical models are based, is thus a single value for each key-participant combination (3 values per participant, which are not independent but sum = 1). What we then do is calculate the difference in the probability of choosing the key with a medium (or high) probability of effect versus the key with a low probability of effect. The result of the reduced probability of the autistic group to choose the medium probability of neutral effect key is based on this variable. We take the reviewer's suggestion and further investigate the homogeneity of the variability between groups in this specific effect of interest (and significant from the models). To do so, we conducted the Levene's test on the aforementioned variable (difference in the probability of choosing the medium probability of effect key versus the low probability of effect key). 

The result is not significant (F= 3.8383; p= 0.052) and does not allow us to reject the null hypothesis of equivalent variance between groups.

R: Since you provided the data I had a quick look at the elephant in the room, namely whether the difference in RT can be explained by the gender differences. Your ASD sample has mostly males whereas your control group is more even, and not as skewed in the gender distribution as the ASD sample is. In a model with RT as outcome variable and gender as predictor I got a significant effect (can't nest within group as one group had no females, e.g. older children). I would be interested to see a model with gender and whether it wins over model 1 and 2.

A: We are aware that our sample is not balanced by gender and we have now explicitly mentioned this as a limitation in representing the general population. In the case of autism, this reflects the male prevalence of the condition, and we have given preference to recruiting male participants for the non-autistic group as well. We did not control for gender in our models not only in light of the unbalanced sample, but also because we had no gender-related research objectives and hypotheses. We preferred to keep the models as simple as possible, prioritising the factors of interest for our research questions. However, as pointed out in the discussion, the models explain a limited amount of variability in the data, and additional intervening variables (including individual differences that might include gender) will need to be explored. However, we do not feel comfortable including analysis on gender effects post hoc, which could lead to misleading conclusions.

R: Table 1 also has for non-autistic adults n=24 (6:8) but 6+8 = 14, is it 6:18 which would be a good match to the autistic group or 16:8?

A: We thank the reviewer for spotting this typo, which has now been corrected (i.e. 6:18).

R: Finally, I missed a conclusion. You have a lengthy section on future perspectives. This is fine but the reader does not know what the take home message is.

A: We agree with the reviewer and thank them for the suggestion. We have included a short conclusion emphasising the take home message.

“The Control-Based Response Selection theory (CBRS) proposes that the mere action-effectiveness facilitates action selection and speed when people make free choices among options. The model hypothesises that motivation by this sense of control often disregards the valence of the effect of one’s action and may be atypical in people who exhibit repetitive behaviours such as stereotypies, which are often present in autism. Our results did not show this agency effect in facilitating the free choices of non-autistic or autistic children and adults. We did, however, find such facilitation for options with a higher probability of resulting in positive effects (reward). In the group of participants with autism, we found signs of reduced tolerance of uncertainty (more infrequent choice of options with a more uncertain outcome), shorter response times (interpretable as a marker of less planning and control of the choice) and greater variability in action processes. Further studies are needed to investigate the neurodiverse mechanisms and individual differences of choice-making in autism, to understand the origins and functions of repetitive behaviours, facilitate flexibility and leveraging self-determination.”

Journal Requirements:

1. Please ensure that your manuscript meets PLOS ONE's style requirements, including those for file naming. The PLOS ONE style templates can be found at 

https://journals.plos.org/plosone/s/file?id=wjVg/PLOSOne_formatting_sample_main_body.pdf and 

https://journals.plos.org/plosone/s/file?id=ba62/PLOSOne_formatting_sample_title_authors_affiliations.pdf

2. We note that the grant information you provided in the ‘Funding Information’ and ‘Financial Disclosure’ sections do not match. When you resubmit, please ensure that you provide the correct grant numbers for the awards you received for your study in the ‘Funding Information’ section.

A: the correct information is as follows:

Funding information: The study was funded by the University of Padova with a SID grant, and by the Beneficentia Stiftung Foundation.

3. We note that you have stated that you will provide repository information for your data at acceptance. Should your manuscript be accepted for publication, we will hold it until you provide the relevant accession numbers or DOIs necessary to access your data. If you wish to make changes to your Data Availability statement, please describe these changes in your cover letter and we will update your Data Availability statement to reflect the information you provide.

A: Data and analysis script are available from the OSF public repository: https://osf.io/skhv6/?view_only=3d8ed47d895b49319ec503ebb39b6d24

4. Please upload a new copy of Figure 6 as the detail is not clear. Please follow the link for more information: https://blogs.plos.org/plos/2019/06/looking-good-tips-for-creating-your-plos-figures-graphics/" https://blogs.plos.org/plos/2019/06/looking-good-tips-for-creating-your-plos-figures-graphics/"

A: Figure 6 has been improved as suggested.

Attachment Submitted filename: Response to Reviewers.pdf Click here for additional data file.

# Editor report: Decision Letter 1

**DOI:** 10.1371/journal.pone.0284407.r003

**Authors:** Yukiori Goto

31 Mar 2023

Agency and reward across development and in autism: a free-choice paradigm

PONE-D-23-02212R1

Dear Dr. Farroni,

We’re pleased to inform you that your manuscript has been judged scientifically suitable for publication and will be formally accepted for publication once it meets all outstanding technical requirements.

Within one week, you’ll receive an e-mail detailing the required amendments. When these have been addressed, you’ll receive a formal acceptance letter and your manuscript will be scheduled for publication.

An invoice for payment will follow shortly after the formal acceptance. To ensure an efficient process, please log into Editorial Manager at http://www.editorialmanager.com/pone/, click the 'Update My Information' link at the top of the page, and double check that your user information is up-to-date. If you have any billing related questions, please contact our Author Billing department directly at authorbilling@plos.org.

If your institution or institutions have a press office, please notify them about your upcoming paper to help maximize its impact. If they’ll be preparing press materials, please inform our press team as soon as possible -- no later than 48 hours after receiving the formal acceptance. Your manuscript will remain under strict press embargo until 2 pm Eastern Time on the date of publication. For more information, please contact onepress@plos.org.

Kind regards,

Yukiori Goto, Ph.D.

Academic Editor

PLOS ONE

Additional Editor Comments (optional):

All concerns raised by the reviewers were adequately addressed by the revision.

Reviewers' comments:

# Editor report: Acceptance letter

**DOI:** 10.1371/journal.pone.0284407.r004

**Authors:** Yukiori Goto

4 Apr 2023

PONE-D-23-02212R1 

Agency and reward across development and in autism: a free-choice paradigm. 

Dear Dr. Farroni:

I'm pleased to inform you that your manuscript has been deemed suitable for publication in PLOS ONE. Congratulations! Your manuscript is now with our production department. 

If your institution or institutions have a press office, please let them know about your upcoming paper now to help maximize its impact. If they'll be preparing press materials, please inform our press team within the next 48 hours. Your manuscript will remain under strict press embargo until 2 pm Eastern Time on the date of publication. For more information please contact onepress@plos.org.

If we can help with anything else, please email us at plosone@plos.org. 

Thank you for submitting your work to PLOS ONE and supporting open access. 

Kind regards, 

PLOS ONE Editorial Office Staff

on behalf of

Dr. Yukiori Goto 

Academic Editor

PLOS ONE
